# Association of Oliguria With Acute Kidney Injury Diagnosis, Severity Assessment, and Mortality Among Patients With Critical Illness

**DOI:** 10.1001/jamanetworkopen.2021.33094

**Published:** 2021-11-04

**Authors:** Nathan Axel Bianchi, Louis Léon Stavart, Marco Altarelli, Tatiana Kelevina, Mohamed Faouzi, Antoine Guillaume Schneider

**Affiliations:** 1 Adult Intensive Care Unit, Centre Hospitalier Universitaire Vaudois, Lausanne, Switzerland; 2 Faculty of Biology and Medicine, University of Lausanne, Lausanne, Switzerland; 3 Division of Biostatistics, Center for Primary Care and Public Health (Unisanté), University of Lausanne, Lausanne, Switzerland

## Abstract

**Importance:**

The current definition and staging of acute kidney injury (AKI) considers alterations in serum creatinine (sCr) level and urinary output (UO). However, the relevance of oliguria-based criteria is disputed.

**Objective:**

To determine the contribution of oliguria, as defined by the Kidney Disease: Improving Global Outcomes (KDIGO) criteria, to AKI diagnosis, severity assessment, and short- and long-term outcomes.

**Design, Setting, and Participants:**

This cohort study included adult patients admitted to a multidisciplinary intensive care unit from January 1, 2010, to June 15, 2020. Patients receiving long-term dialysis and those who declined consent were excluded. Daily sCr level and hourly UO measurements along with sociodemographic characteristics and severity scores were extracted from electronic medical records. Long-term mortality was assessed by cross-referencing the database with the Swiss national death registry. The onset and severity of AKI according to the KDIGO classification was determined using UO and sCr criteria separately, and their agreement was assessed.

**Main Outcomes and Measures:**

Using a multivariable model accounting for baseline characteristics, severity scores, and sCr stages, the association of UO criteria with 90-day mortality was evaluated. Sensitivity analyses were conducted to assess how missing sCr, body weight, and UO values, as well as different sCr baseline definitions and imputations methods, would affect the main results.

**Results:**

Among the 15 620 patients included in the study (10 330 men [66.1%] with a median age of 65 [IQR, 53-75] years, a median Simplified Acute Physiology Score II score of 40.0 [IQR, 30.0-53.0], and a median follow-up of 67.0 [IQR, 34.0-100.0] months), 12 143 (77.7%) fulfilled AKI criteria. Serum creatinine and UO criteria had poor agreement on AKI diagnosis and staging (Cohen weighted κ, 0.36; 95% CI, 0.35-0.37; *P* < .001). Compared with the isolated use of sCr criteria, consideration of UO criteria enabled identification of AKI in 5630 patients (36.0%). Those patients had a higher 90-day mortality than patients without AKI (724 of 5608 [12.9%] vs 288 of 3462 [8.3%]; *P* < .001). On multivariable analysis accounting for sCr stage, comorbidities, and illness severity, UO stages 2 and 3 were associated with a higher 90-day mortality (odds ratios, 2.4 [95% CI, 1.6-3.8; *P* < .001] and 6.2 [95% CI, 3.7-10.5; *P* < .001], respectively). These results remained significant in all sensitivity analyses.

**Conclusions and Relevance:**

The findings of this cohort study suggest that oliguria lasting more than 12 hours (KDIGO stage 2 or 3) has major AKI diagnostic implications and is associated with outcomes irrespective of sCr elevations.

## Key Points

**Question:**

What is the contribution of the oliguria-based Kidney Disease: Improving Global Outcomes (KDIGO) criteria to diagnosis, severity assessment, and outcomes in acute kidney injury (AKI)?

**Findings:**

In this cohort study of 15 620 patients admitted to an intensive care unit, consideration of oliguria-based criteria, in addition to those based on serum creatinine levels alone, enabled identification of AKI or an increase in its severity in 51.7% of critically ill patients. Moreover, oliguria-based stages 2 and 3 AKI appeared to be statistically significantly associated with 90-day mortality after correction for serum creatinine level, comorbidities, and illness severity.

**Meaning:**

These findings suggest that oliguria, as defined by the KDIGO criteria, is associated with mortality in AKI and should be accounted for by clinicians at the bedside and clinical researchers while designing trials.

## Teaser

This cohort study examines the association of oliguria with diagnosis, severity assessment, and short- and long-term outcomes of acute kidney injury among patients admitted to an adult intensive care unit.

## Introduction

Acute kidney injury (AKI) has been proposed to encompass the entire range of acute alterations in kidney function, from transient oliguria to severe loss of function.^1^ According to the Kidney Disease: Improving Global Outcomes (KDIGO) guidelines, AKI is defined as an increase in serum creatinine (sCr) levels relative to a baseline value and/or a decrease in weight-adjusted hourly urinary output (UO).^2^ Both components are deemed readily available at the bedside, are reproducible, and are assumed to have equivalent prognostic capacities.

The added value of oliguria over sCr level, however, is challenged,^3^ particularly if it is only transient and not associated with an increase in sCr level.^4^ Most studies reporting AKI outcomes have solely relied on sCr criteria or have applied modified/simplified UO criteria.^2,5,6,7,8,9^ Indeed, sCr levels are regularly monitored in hospitalized patients and can easily be retrieved through electronic medical records. Hence, sCr-based staging is straightforward and can even trigger bedside e-alerts.^10^ In contrast, KDIGO criteria for oliguria are relatively complex and cumbersome. They require hourly UO monitoring, the presence of an indwelling catheter, and reconciling UO documentation with precise time frames (6, 12, and 24 hours).^11^ Furthermore, UO criteria rely on the correct estimation or documentation of patients’ body weight, a notoriously poorly documented parameter.^12^

To the best of our knowledge, only a handful of studies^6,7,13,14^ have assessed the influence of oliguria, defined by nonmodified KDIGO criteria, on AKI diagnosis, severity assessment, and patient outcomes. A large single-center retrospective study (32 045 critically ill patients)^6^ suggested that UO and sCr criteria contributed differently to AKI diagnosis and appeared to be additive. Unfortunately, the authors were not able to analyze the burden of many confounding factors and did not express the importance of reclassification by UO criteria. Their conclusions were somewhat confirmed by a multicenter retrospective study conducted in China.^7^ In that study, oliguria-based AKI was independently associated with higher in-hospital mortality rates. However, the study included a relatively limited number of patients (N = 1058), and patients’ follow-up was limited to 3 months. In a retrospective study of 6637 patients undergoing cardiac surgery,^13^ consideration of UO criteria increased the incidence of AKI from 38.6% to 81.2%. Similarly, an AKI diagnosis would have been missed in 67.5% of cases if only sCr levels had been considered in a cohort of children and young adults.^14^ However, these studies focused on very specific populations, and therefore the real impact of oliguria-based criteria on AKI classification and outcomes among critically ill patients remains unclear.^11^ Accordingly, we designed a large cohort study of critically ill patients admitted to our institution’s adult intensive care unit (ICU) to determine the association of oliguria, as defined by KDIGO criteria, with AKI diagnosis, severity assessment, and patients’ short- and long-term outcomes.

## Methods

### Ethics

This study was approved by the Ethics Committee of the Canton of Vaud. In accordance with the Swiss Federal Act on Research Involving Human Beings (article 34),^15^ retrospective use of nongenetic health-related personal data was permitted, provided that the patient (or his or her legal representative) had not expressed wishes of not participating in clinical research. This study followed the Strengthening the Reporting of Observational Studies in Epidemiology (STROBE) reporting guideline.

### Patient Selection

All adult (aged ≥18 years) patients admitted for more than 6 hours within our tertiary ICU from January 1, 2010, to June 15, 2020, were considered for inclusion. Those with (1) documented or expressed wishes of nonparticipation in clinical research, (2) end-stage kidney disease and receiving long-term kidney replacement therapy (KRT), or (3) incomplete ICU data precluding AKI definition and staging (ie, <6 hours of UO measurements, or absence of sCr measurement during the ICU stay) were excluded. For patients with multiple admissions, only the first admission was considered.

### Data Collection

Data were extracted from electronic medical records (MetaVision [IMD Soft] and Soarian [Cerner]). We collected patients’ characteristics (age, sex, weight, height, and nationality), admission status (elective or emergency), movements (pre-ICU location, hospital and ICU length of stay, and discharge destination), underlying medical conditions (defined by the *International Statistical Classification of Diseases and Related Health Problems, Tenth Revision*),^16^ ICU main diagnoses, and interventions (mechanical ventilation and KRT). We also computed the Charlson Comorbidity Index, the Simplified Acute Physiology Score (SAPS) II, and a modified version of the SAPS II score excluding the UO parameter.^17,18^ In addition, to enable AKI classification, all sCr measurements obtained during the patient’s ICU stay and within the 12 months preceding ICU admission were collected. Similarly, all hourly UO measurements obtained during the patient’s ICU stay were recorded.

### Definitions

#### Reference Baseline sCr Level

The reference baseline sCr level was defined as the lowest sCr level recorded within 12 months of ICU admission. Values measured within 24 hours of unscheduled admissions were excluded because they were deemed more likely to represent acute conditions rather than a baseline value. In the absence of eligible sCr levels measured before ICU admission, we considered the lowest value available during the ICU stay after excluding values measured during KRT and within an arbitrary period of 3 days after KRT. In addition, to account for the temporality inherent to KDIGO sCr criteria (see next section), 48-hour and 7-day sliding windows were used to define dynamic baselines.

#### AKI Diagnosis and Staging According to sCr Criteria

According to KDIGO criteria, AKI was defined as an increase in sCr level to at least 1.5 times the baseline value within a 7-day period, or an increase in sCr level by at least 0.3 mg/dL (to convert to μmol/L, multiply by 88.4) within a 48-hour time frame. Stage 1 was defined as a peak to baseline difference of at least 0.3 mg/dL or a peak to baseline ratio of 1.5 to 1.9; stage 2, by a peak to baseline ratio of 2.0 to 2.9; and stage 3, by a peak to baseline ratio of at least 3.0, an sCr level of at least 4.0 mg/dL, or the receipt of KRT. Staging was considered only if time rules of AKI definition were fulfilled.^2^

#### UO Assessment

For every single hour of each ICU stay, we computed a 6-hour mean corresponding to the mean UO measured within the previous 6 hours. Similarly, we calculated a 12-hour mean (mean UO measured within the previous 12 hours) and a 24-hour mean (mean UO within the previous 24 hours). All values were adjusted to the patient’s body weight (units in mL/kg/h). By definition, the 6-hour mean hourly UO values could not have been computed for the first 5 hours of ICU admission; the 12-hour mean hourly OU values, for the first 11 hours of ICU admission; and the 24-hour mean hourly UO values, for the first 23 hours of ICU admission. Therefore, AKI could not have been diagnosed using UO criteria before 6 hours of ICU stay.

#### AKI Diagnosis and Staging According to UO

According to the KDIGO criteria, AKI was defined, on an hourly basis, as a 6-hour mean of less than 0.5 mL/kg/h. An AKI of stage 1 was defined as a 6-hour mean of less than 0.5 mL/kg/h, stage 2, as a 12-hour mean of less than 0.5 mL/kg/h; and stage 3, as a 24-hour mean of less than 0.3 mL/kg/h or a 12-hour mean equal to 0 mL/kg/h (anuria). As implied by such definitions, AKI stage 2 or 3 could not have been diagnosed using UO criteria before 12 hours of ICU stay.

### Overall AKI Diagnosis and Group Allocation

An AKI stage was attributed to each patient for every single hour of the ICU stay based on (1) sCr criteria, (2) UO criteria, and (3) the highest among sCr and UO criteria (overall stage). For diagnosis and classification, we considered the highest AKI stage reached by a patient using sCr criteria (sCr stage), UO criteria (UO stage), and overall (overall stage) at any time within the ICU stay. Finally, patients were classified into 1 of the following categories: no AKI if they never fulfilled any AKI criteria; sCr plus UO if they fulfilled both criteria; UO only if they fulfilled UO but not sCr criteria; or sCr only if they fulfilled sCr but not UO criteria.

### Missing Values Management

For missing sCr values, we attributed values calculated by linear approximation between the previous and the next measures available. When only 1 value was available, a similar value was attributed to the previous day or the next day. For missing UO values, we attributed values calculated by dividing the next available value by the number of adjacent missing values (eg, the sequence missing, missing, missing, 200 was replaced with 50, 50, 50, 50), assuming the sequence corresponded to consolidated data entry.

Missing values at the end of the ICU stay were not considered. An hourly UO value greater than 1000 mL that remained after processing of missing values was considered erroneous and replaced by the closest hourly UO value available. The number of missing and attributed values are reported below.

### Body Weight

We primarily considered the documented preadmission body weight. In the absence of such a value, we used the first quartile of all weights measured throughout the ICU stay (after exclusion of values <30 kg). If none of these values were available, arbitrary body weights of 60 and 70 kg were attributed to women and men, respectively.

### Outcomes

Our primary outcome was 90-day mortality. The patients’ vital status was assessed by cross-referencing our hospital database with the Swiss national death registry. Mortality and time to death were recorded. Follow-up was pursued until August 21, 2020, or the patient’s death. Secondary outcomes were ICU, in-hospital, 1-year, 3-year, and 5-year mortality rates, ICU and hospital length of stay, and the need for mechanical ventilation and KRT during the ICU stay.

### Statistical Analysis

Continuous data were reported as mean (SD) or median (IQR). Continuous data were compared using unpaired *t* tests or analysis of variance and Mann-Whitney or Kruskal-Wallis test according to the underlying data distribution. Categorical variables were expressed as number (percentage) and compared using a χ^2^ test or a Fisher exact test. The agreement between sCr and UO criteria was assessed by Cohen unweighted and quadratic κ coefficients. We used the Kaplan-Meier method to estimate the overall and groups’ 90-day survival function and log-rank regressions to assess group differences. Logistic regressions were used to test the association between the 90-day mortality and risk factors. The strength of the association was measured using odds ratios (ORs), *P* values, and 95% CIs. The functional relationship (linearity assumption) between continuous variables and the outcome was assessed using fractional polynomials. Univariate logistic regression models were performed to (1) assess the association of overall KDIGO stages with 90-day mortality and (2) separately assess the association of sCr and UO criteria with 90-day mortality. A multivariate logistic regression analysis was performed to assess the association of UO criteria with 90-day mortality after correction for AKI stage according to sCr criteria and for various other cofactors. Significant cofactors (*P* < .05) (eg, demographics, admission status, complications, comorbidities, Charlson Comorbidity Index, and modified SAPS II scores) were selected in the model using a stepwise backward approach. Potential interactions were tested. Collinearity was assessed using variance inflation factors. Only variables with variance inflation factors of less than 5 were included in the final model. Model diagnostics were performed using standard tools for logistic regression models, and the goodness of fit was tested using Hosmer-Lemeshow test. Power discrimination between groups (dead vs alive) was assessed using the area under the receiver operator characteristics curve.

Sensitivity analyses were performed to explore the impact of missing or imputed values of (1) baseline sCr, (2) body weight, (3) daily sCr, and (4) hourly UO on our model. Accordingly, we tested in our model the interaction of the following dichotomic subgroups variables: (1) baseline sCr value (known vs inferred), (2) body weight (known vs inferred), (3) absence of imputed daily sCr values (yes vs no), and (4) less than 5.4% of imputed hourly UO values (yes vs no). The latter percentage (5.4%) corresponds to the median percentage of missing hourly UO values among all study patients.

Additional sensitivity analyses were performed to assess how alternate baseline sCr definitions affected our model. We considered the following alternate definitions: (1) mean sCr level within 365 days of ICU admission, (2) mean sCr level within 365 days after exclusion of values obtained within 24 hours of ICU admission, and (3) mean sCr level within 365 days after exclusion of values obtained within 7 days of ICU admission. In addition, we repeated our analyses assuming a normal (75 mL/min/1.73 m^2^) estimated glomerular filtration rate for patients for whom no baseline sCr level was available. Finally, we also repeated the multivariate logistic regression analysis after excluding the modified SAPS II score.

For all analyses, a 2-tailed *P* < .05 was considered statistically significant, except for the multiple outcomes comparisons where a Bonferroni correction was applied. Statistical analyses were performed with R, version 4.1.0 (including extension packages survival, survminer, tableone, and vcd [R Core Team]), and STATA, version 16 (StataCorp LLC).

## Results

### Population

During the study period, 18 134 patients were admitted to our ICU. A total of 2514 patients were excluded, including 1116 who declined institutional consent for data reuse, 84 younger than 18 years, 747 with an ICU stay of less than 6 hours, 217 undergoing long-term KRT for end-stage kidney disease, 45 who could not be matched with administrative data, and 305 with incomplete data precluding AKI definition and staging. Therefore, we included 15 620 patients in our main analysis. Their demographic and clinical characteristics at ICU admission are presented in Table 1. A total of 10 330 patients (66.1%) were male and 5290 (33.9%) were female; their median age at ICU admission was 65 (IQR, 53-75) years. Data on race and ethnicity are not recorded in the electronic medical record and could therefore not be extracted. Median SAPS score was 40 (IQR, 30-53), and median follow-up was 67 (IQR, 34-100) months.

**Table 1.** Patient Demographics and Characteristics at ICU Admission

Characteristic	Study group^a^				
	All (N = 15 620)	No AKI (n = 3477)	AKI according to KDIGO criteria (n = 12 143)		
			sCr plus UO (n = 5524)	UO only (n = 5630)	sCr only (n = 989)
Demographic					
Age at ICU admission, median (IQR), y	65.0 (53.0-75.0)	60.0 (47.0-71.0)	67.0 (56.0-76.0)	66.0 (55.0-75.0)	61.0 (47.0-72.0)
Sex					
Male	10 330 (66.1)	2209 (63.5)	3745 (67.8)	3710 (65.9)	666 (67.3)
Female	5290 (33.9)	1268 (36.5)	1779 (32.2)	1920 (34.1)	323 (32.7)
Body weight, median (IQR), kg					
Actual^b^	75.0 (65.0-87.0)	71.0 (62.0-80.0)	79.0 (66.0-90.0)	77.0 (66.0-88.0)	70.0 (60.0-80.0)
Considered^c^	72.0 (65.0-84.0)	70.0 (60.0-77.0)	75.0 (65.2-87.0)	74.0 (66.0-85.0)	70.0 (60.0-79.4)
BMI, median (IQR)^d^	25.7 (22.9-29.1)	24.2 (21.6-26.7)	26.2 (23.4-30.1)	26.0 (23.4-29.4)	24.1 (20.9-27.3)
Kidney function parameters					
Baseline sCr level, median (IQR), mg/dL					
Actual^e^	0.8 (0.7-1.0)	0.8 (0.7-1.0)	0.9 (0.7-1.1)	0.9 (0.7-1.0)	0.8 (0.6-1.0)
Considered^f^	0.8 (0.6-1.0)	0.8 (0.6-1.0)	0.8 (0.6-1.1)	0.8 (0.7-1.0)	0.7 (0.5-1.0)
Baseline eGFR,^g^ mL/min/1.72 m^2^					
>60	6785 (84.0)	1619 (90.5)	2027 (76.4)	2725 (86.8)	414 (83.6)
30-60	1137 (14.1)	154 (8.6)	523 (19.7)	391 (12.5)	69 (13.9)
<30	151 (1.9)	15 (0.8)	102 (3.8)	22 (0.7)	12 (2.4)
SAPS II, median (IQR)^h^	40.0 (30.0-53.0)	31.0 (23.0-40.0)	51.0 (40.0-65.0)	36.0 (28.0-45.0)	43.0 (33.0-53.0)
Coexisting conditions					
Charlson Comorbidity Index, median (IQR)	4.0 (2.0-6.0)	3.0 (1.0-5.0)	5.0 (3.0-7.0)	4.0 (2.0-6.0)	4.0 (2.0-7.0)
Hypertension	7318 (47.0)	1325 (38.4)	2852 (51.7)	2744 (48.8)	397 (40.)
Diabetes	2939 (18.9)	473 (13.7)	1287 (23.3)	975 (17.4)	204 (20.7)
Chronic kidney disease	1619 (10.4)	175 (5.1)	941 (17.1)	349 (6.2)	154 (15.7)
Ischemic heart disease	5454 (35.0)	1202 (34.9)	1862 (33.7)	2165 (38.5)	225 (22.9)
Chronic heart failure	3471 (22.3)	485 (14.1)	1741 (31.5)	1033 (18.4)	212 (21.5)
Cancer	2914 (18.7)	577 (16.7)	1121 (20.3)	967 (17.2)	249 (25.3)
COPD	1811 (11.6)	322 (9.3)	777 (14.1)	591 (10.5)	121 (12.3)
Chronic liver disease	1192 (7.7)	156 (4.5)	647 (11.7)	284 (5.1)	105 (10.7)
Type of admission					
Surgical	8724 (57.2)	1923 (56.6)	2888 (53.7)	3488 (63.3)	425 (44.0)
Medical	6536 (42.8)	1476 (43.4)	2492 (46.3)	2026 (36.7)	542 (56.0)
Elective admission	4524 (29.6)	1187 (34.9)	978 (18.2)	2242 (40.7)	117 (12.1)
sCr level at ICU admission, median (IQR), mg/dL	0.9 (0.7-1.3)	0.8 (0.7-1.0)	1.3 (0.9-1.8)	0.8 (0.7-1.0)	1.2 (0.9-1.8)
Interventions within first 24 h					
Mechanical ventilation	9196 (58.9)	1612 (46.4)	3979 (72.0)	3104 (55.1)	501 (50.7)
Noradrenaline	10 754 (68.8)	1833 (52.7)	4610 (83.5)	3655 (64.9)	656 (66.3)
Main ICU diagnosis					
Cardiovascular surgery	3505 (23.1)	867 (25.5)	885 (16.5)	1684 (30.6)	69 (7.2)
Other acute cardiopathies	1964 (12.9)	587 (17.3)	525 (9.8)	762 (13.9)	90 (9.4)
Septic shock	1762 (11.6)	181 (5.3)	1044 (19.5)	297 (5.4)	240 (25.0)
Neurological	1615 (10.6)	456 (13.4)	380 (7.1)	667 (12.1)	112 (11.7)
Acute respiratory insufficiency	1314 (8.6)	247 (7.3)	492 (9.2)	494 (9.0)	81 (8.4)
Trauma	1237 (8.1)	279 (8.2)	468 (8.7)	415 (7.5)	75 (7.8)
Cardiopulmonary arrest	899 (5.9)	73 (2.1)	557 (10.4)	225 (4.1)	44 (4.6)
Other	2909 (19.1)	706 (20.8)	998 (18.7)	956 (17.4)	249 (25.9)

Abbreviations: AKI, acute kidney injury; BMI, body mass index; COPD, chronic obstructive pulmonary disease; eGFR, estimated glomerular filtration rate; ICU, intensive care unit; KDIGO, Kidney Disease: Improving Global Outcomes; SAPS, Simplified Acute Physiology Score; sCr, serum creatinine; UO, urinary output.

SI conversion factor: To convert sCr to μmol/L, multiply by 88.4.

^a^ Unless otherwise indicated, data are expressed as number (%) of patients. Percentages have been rounded and may not total 100. Owing to missing data, denominators may not total numbers in column headings. A detailed description of missing values management can be found in the Methods section.

^b^ Body weight available for 12 414 (79.5%) of all patients.

^c^ Considered body weight for AKI diagnosis and staging after imputation of missing values (see Methods section).

^d^ Calculated as actual weight in kilograms divided by actual height in square meters for 65.9% of all patients.

^e^ Available for 8073 (51.7%) of all patients.

^f^ Considered baseline sCr level for AKI diagnosis and staging after imputation of missing values (see Methods section).

^g^ Estimated using the modification of diet in kidney disease equation based on actual baseline sCr level and patient age and sex (all patients were considered White).

^h^ Scores range from 0 to 163, with higher scores indicating higher in-hospital mortality risks.

### AKI Incidence, Severity, and Association With 90-Day Mortality

As shown in Table 1, according to the KDIGO definition (overall stage), 12 143 patients (77.7%) presented with at least 1 episode of AKI during their ICU stay. The maximum AKI severity was stage 1 in 3027 patients (19.4%), stage 2 in 6329 patients (40.5%), and stage 3 in 2787 patients (17.8%) (Table 2).

**Table 2.** 90-Day Mortality According to AKI Stage by Kidney Disease: Improving Global Outcomes Criteria

	Patients, No. (%)	Mortality, No./total No. (%)^b^	Univariate analysis^a^		
			OR (95% CI)	*P* value
Overall AKI stage				
No AKI	3477 (22.3)	288/3462 (8.3)	1 [Reference]	NA
Stage 1	3027 (19.4)	452/3013 (15.0)	1.94 (1.66-2.27)	<.001
Stage 2	6329 (40.5)	1074/6304 (17.0)	2.26 (1.97-2.59)	<.001
Stage 3	2787 (17.8)	1045/2772 (37.7)	6.67 (5.78-7.69)	<.001
AKI stage according to UO criteria^c^				
No AKI	4466 (28.6)	462/4444 (10.4)	1 [Reference]	NA
Stage 1	2646 (16.9)	410/2635 (15.6)	1.59 (1.38-1.83)	<.001
Stage 2	6390 (40.9)	1120/6365 (17.6)	1.84 (1.64-2.06)	<.001
Stage 3	2118 (13.5)	867/2107 (41.1)	6.03 (5.29-6.86)	<.001
AKI stage according to sCr criteria^d^				
No AKI	9107 (58.3)	1012/9070 (11.2)	1 [Reference]	NA
Stage 1	3422 (21.9)	747/3406 (21.9)	2.23 (2.01-2.48)	<.001
Stage 2	1238 (7.9)	334/1233 (27.1)	2.95 (2.57-3.40)	<.001
Stage 3	1853 (11.9)	766/1842 (41.6)	5.67 (5.06-6.34)	<.001

Abbreviations: AKI, acute kidney injury; NA, not applicable; OR, odd ratio; sCr, serum creatinine; UO, urinary output.

^a^ Unadjusted ORs for 90-day mortality per AKI severity stage according to KDIGO criteria by univariate logistic regression analysis. Area under the receiver operating characteristics curve was 0.66 for overall AKI stage, 0.64 for AKI stage by UO criteria, and 0.66 for AKI stage by sCr criteria.

^b^ Indicates crude 90-day mortality. Vital status at 90 days was available for 15 551 patients (99.5%). Percentages are shown for patients with available vital status at 90 days.

^c^ Irrespective of sCr level.

^d^ Irrespective of UO.

Patients with AKI had a higher 90-day mortality than those without AKI (2571 of 12 089 [21.3%] vs 288 of 3462 [8.3%]; *P* < .001). Among patients with AKI, 90-day mortality increased with severity: it was 15.0% (452 of 3013) in patients with AKI stage 1, 17.0% (1074 of 6304) in those with AKI stage 2, and 37.7% (1045 of 2772) in those with AKI stage 3 (*P* < .001 for all) (Table 2).

### Relative Contribution of UO and sCr Criteria to AKI Diagnosis

Both UO and sCr criteria were met in 5524 patients (35.4%) (sCr plus UO group). The UO criteria (but not sCr) were met in 5630 patients (36.0%) (UO-only group), whereas the sCr criteria (but not UO) were met in 989 patients (6.3%) (sCr-only group).

#### Agreement Between sCr and UO Criteria for AKI Staging

**Figure 1.**
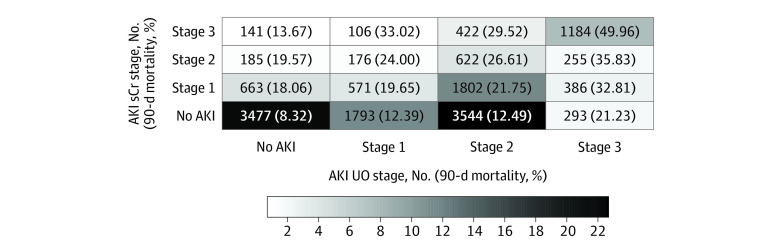
Classification of Patients and Corresponding 90-Day Mortality According to the Worst Kidney Disease: Improving Global Outcomes Serum Creatinine (sCr) and Urinary Output (UO) Stages Data represent the number of patients fulfilling UO and sCr criteria at some stage during their intensive care unit stay and their 90-day mortality rate. The density scale represents the percentage of all included patients within a given category. The agreement between sCr and UO criteria was poor (weighted κ coefficient, 0.36; 95% CI, 0.35-0.37; *P* < .001). AKI indicates acute kidney injury.

Similar AKI stage was reached by both sCr and UO criteria in 5854 patients (37.5%) (Figure 1). The agreement between both criteria for AKI staging was poor (κ, 0.16; 95% CI, 0.15-0.17; *P* < .001) and remained fair while using quadratic weighted κ coefficients to account for degree of disagreement (κ, 0.36; 95% CI, 0.35-0.37; *P* < .001).

#### UO Reclassification Rate

Compared with staging based on the sole sCr criteria, consideration of UO criteria enabled us to reclassify AKI in 8073 patients (51.7%). This included 5630 patients whose AKI occurrence was only demonstrated by UO criteria (UO-only group) and 2443 patients whose AKI severity was increased by UO criteria (Figure 1). The UO-only group notwithstanding, AKI stage was upgraded by 1 stage in 2057 patients (13.2% of all included patients) and 2 stages in 386 patients (2.5% of all included patients).

### Association of UO With 90-Day Mortality

#### Outcomes of Patients in the UO-Only Group

Patients in the UO-only group had a higher 90-day mortality in comparison with patients without AKI (724 of 5608 [12.9%] vs 288 of 3462 [8.3%]; *P* < .001). Those results persisted at the 1-year (930 of 5224 [17.8%] vs 429 of 3243 [13.2%]; *P* < .001), 3-year (1088 of 4153 [26.2%] vs 529 of 2618 [20.2%]; *P* < .001), and 5-year (996 of 3175 [31.4%] vs 482 of 2007 [24.0%]; *P* < .001) follow-ups (Table 3 and eTable 1 in the Supplement). On the other hand, patients in the UO-only group had lower mortality than those in the sCr-only group (eg, 90-day mortality, 724 of 5608 [12.9%] vs 174 of 982 [17.7%]; *P* < .001) and sCr plus UO group (eg, 90-day mortality, 724 of 5608 [12.9%] vs 1673 of 5499 [30.4%]; *P* < .001). (Table 3 and eTable 1 in the Supplement). Overall survival probabilities for each group are presented in eFigure 1 in the Supplement.

**Table 3.** Patient Outcomes

Outcome	Patient group^a^				
	All (N = 15 620)	No AKI (n = 3477)	AKI according to KDIGO criteria (n = 12 143)		
			sCr plus UO (n = 5524)	UO only (n = 5630)	sCr only (n = 989)
LOS, median (IQR), d					
ICU	2.3 (1.1-5.6)	1.1 (0.9-1.9)	5.8 (2.8-12.3)	1.9 (1.0-3.3)	2.8 (1.5-5.5)
Hospital	12.8 (6.8-23.4)	9.2 (4.0-15.8)	18.9 (9.2-34.8)	11.1 (6.5-18.2)	14.4 (7.7-26.2)
KRT during ICU stay	1160 (7.4)	0	1130 (20.5)	0	30 (3.0)
Mechanical ventilation	9640 (61.7)	1624 (46.7)	4335 (78.5)	3168 (56.3)	513 (51.9)
Mechanical ventilation total duration, median (IQR), h	35.7 (10.6-122.2)	10.1 (5.0-22.1)	99.6 (35.5-231.4)	18.2 (7.2-48.8)	46.5 (15.4-128.8)
Mortality					
ICU	1723 (11.0)	147 (4.2)	1129 (20.4)	379 (6.7)	68 (6.9)
Hospital	2306 (14.8)	202 (5.8)	1452 (26.3)	534 (9.5)	118 (11.9)
90-d^b^	2859 (18.4)	288 (8.3)	1673 (30.4)	724 (12.9)	174 (17.7)
1-y^c^	3453 (23.9)	429 (13.2)	1858 (36.7)	930 (17.8)	236 (26.0)
3-y^d^	3630 (31.6)	529 (20.2)	1748 (43.8)	1088 (26.2)	265 (36.0)
5-y^e^	3192 (36.9)	482 (24.0)	1478 (50.1)	996 (31.4)	236 (44.7)

Abbreviations: AKI, acute kidney injury; ICU, intensive care unit; KDIGO, Kidney Disease: Improving Global Outcomes; KRT, kidney replacement therapy; LOS, length of stay; sCr, serum creatinine; UO, urinary output.

^a^ Unless otherwise indicated, data are expressed as number (%) of patients. Percentages have been rounded and may not total 100. One-to-one comparisons are reported in eTable 1 in the Supplement.

^b^ Data were available for 15 551 (99.5%) of all patients, 3462 (99.6%) of the no AKI group, 5499 (99.5%) of the sCr plus UO group, 5608 (99.6%) of the UO-only group, and 982 (99.3%) of the sCr-only group.

^c^ Data were available for 14 435 (92.4%) of all patients, 3243 (93.3%) of no AKI group, 5059 (91.6%) of the sCr plus UO group, 5224 (92.8%) of the UO-only group, and 909 (91.9%) of the sCr-only group.

^d^ Data were available for 11 495 (73.6%) of all patients, 2618 (75.3%) of the no AKI group, 3987 (72.2%) of the sCr plus UO group, 4153 (73.8%) of the UO-only group, and 737 (74.5%) of the sCr-only group.

^e^ Data were available for 8662 (55.5%) of all patients, 2007 (57.7%) of the no AKI group, 2952 (53.4%) of the sCr plus UO group, 3175 (56.4%) of the UO-only group, and 528 (53.4%) of the sCr-only group.

#### Association of UO Stage With Mortality

**Figure 2.**
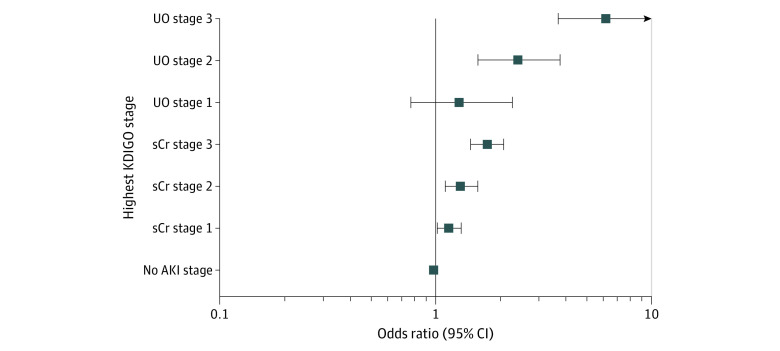
Multivariate Adjusted Odds Ratios for 90-Day Mortality Adjusted odds ratio for 90-day mortality per acute kidney injury (AKI) severity stage according to Kidney Disease: Improving Global Outcomes (KDIGO) serum creatinine (sCr) or urinary output (UO) criteria. The association between AKI severity and 90-day mortality was explored in a multivariate logistic regression model. Variables included in the model were age at intensive care unit admission, baseline sCr level, modified Simplified Acute Physiology Score II, Charlson Comorbidity Index, and main intensive care unit diagnosis. Because no collinearity was found between KDIGO sCr and UO criteria, both were included together in the model. Goodness of fit was assessed by the Hosmer-Lemeshow test (χ^2^_498_ = 430.47; *P* = .99). Discrimination power assessed by the area under the receiver operating characteristic curve for the model for 90-day mortality was 0.87 (95% CI, 0.86-0.87; *P* = .01). A total of 14 852 of 15 551 patients (95.5%) were included in the analysis.

On univariate analysis, AKI severity as assessed by UO criteria (UO stage) was associated with mortality (eg, OR for stage 1 AKI, 1.59; 95% CI, 1.38-1.83) (Table 2). As shown in Figure 1, UO stage appeared to modulate crude 90-day mortality within a given sCr stage; for example, among patients with stage 1 sCr, those with UO stage 3 had higher 90-day mortality (126 of 384 [32.8%]) compared with those with UO stage 2 (390 of 1793 [21.8%]), UO stage 1 (112 of 570 [19.7%]), and no AKI according to UO (119 of 659 [18.1%]). After correction for sCr stage, age, baseline sCr level, Charlson Comorbidity Index, modified SAPS II score, and ICU diagnosis, UO stage 1 was not associated with an increased 90-day mortality (OR, 1.31; 95% CI, 0.76-2.26; *P* = .32). However, UO stage 2 (OR, 2.43; 95% CI, 1.57-3.77; *P* < .001) and UO stage 3 (OR, 6.24; 95% CI, 3.69-10.52; *P* < .001) were both associated with an increased 90-day mortality (Figure 2). Adjusted marginal probabilities of 90-day mortality for each combination of UO and sCr stages derived from the multivariable logistic regression model are presented in eFigure 2 in the Supplement. Within each sCr stage, the additional presence of UO stage was associated with a statistically significant increase in 90-day mortality; for example, among patients with stage 1 sCr, those with UO stage 3 had higher 90-day mortality risks (marginal probability, 0.37) compared to those with UO stage 2 (marginal probability, 0.17), UO stage 1 (marginal probability, 0.16) and no AKI UO (marginal probability, 0.11).

### Sensitivity Analyses

As shown in eTable 2 in the Supplement, the body weight value was missing in 3206 patients (20.5%); baseline sCr value, in 7547 patients (48.3%); any daily sCr value, in 6598 patients (42.2%); and more than 5.4% of hourly UO values, in 7796 patients (49.9%). According to sensitivity analyses, none of these parameters affected our main result (eFigure 3 in the Supplement). Similarly, alternate definitions of baseline sCr or assumption of a normal estimated glomerular filtration rate in case of missing baseline sCr values did not affect our findings (eFigure 4 and 5 in the Supplement, respectively). Finally, exclusion of the modified SAPS II score from the multivariate logistic regression model did not change the main results (eTable 3 in the Supplement).

## Discussion

### Key Findings

We conducted a large cohort study including 15 620 patients admitted to a tertiary ICU. We found that 77.7% of all patients fulfilled KDIGO criteria for AKI at some stage during their ICU stay. There was poor agreement between sCr and UO criteria for AKI diagnosis. Urinary output criteria allowed us to identify AKI in patients who did not fulfill sCr criteria or to upgrade the AKI stage in 51.7% of the patients. Those reclassifications had an important prognostic implication because (1) patients exclusively discovered by UO criteria (no increase in sCr value) had a greater 90-day mortality than those without AKI, and (2) UO stage appeared to modulate the 90-day mortality within each sCr-based AKI stage. Finally, even after correction for sCr stage, comorbidities, and illness severity, UO stages 2 and 3 were strongly associated with 90-day mortality.

### Comparisons With Previous Studies

Consideration of oliguria-based criteria greatly affects the number of patients classified as having AKI.^19^ Nevertheless, many studies^6,7,8,20^ did not consider them or used simplified UO criteria without any concerns about the rigid time frames required for an adequate KDIGO classification. Although high-quality epidemiological studies have actually considered UO-based criteria, the association of UO criteria with diagnosis and outcomes has not been reported.^21,22,23,24,25,26^

To the best of our knowledge, only few large studies^6,7^ have compared the relative diagnostic abilities of oliguria- and sCr-based criteria of the current KDIGO consensus. The first was a large (32 045 patients) retrospective study conducted in a single US center.^6^ The overall incidence of AKI (74.5%) was similar to that reported in the present study. Isolated oliguria was frequently observed and associated with poor 1-year outcomes. Stage 2 AKI only identified by UO also appeared to be the most prevalent group (5421 patients [16.9%]).^6^ Similarly, patients meeting both UO and sCr criteria had a lower survival compared with those who met a single criterion, particularly if the stage met was the same for both criteria. In this study, the importance of patient’s reclassification by UO and its association with outcomes was not explored. Finally, the multivariable analyses only accounted for age and comorbidities.

The second study was a secondary analysis of a prospective multicenter study that included 1058 ICU patients in China.^7^ The relevance of this study was limited by its relatively small number of patients, its short follow-up, and the relative lack of details regarding AKI definition methods. However, similar to our study, the authors also observed a poor agreement between sCr- and UO-based criteria. The rate of reclassifications attributable to UO was much lower (77 patients [7.3%]) than in our study (8073 patients [51.7%]). In that study, the consideration of UO enabled the investigators to define AKI in only 31 patients and to upgrade the severity stage in 46. These discrepancies with our study could be explained by (1) nonstandardized UO assessment and different management of missing values, (2) distinct populations with a higher prevalence of medical patients and respiratory issues, and (3) differences in fluid management leading to different incidence of AKI.^9^ Nevertheless, on multivariate analyses, UO criteria were also found to be an independent risk factor for 90-day mortality (OR, 2.89; 95% CI, 1.96-4.25; *P* < .001).

Finally, 2 studies^13,14^ conducted in specific populations have shown that consideration of UO criteria largely increased the detected incidence of AKI. In both cohorts, the proportion of cases identified by oliguria alone was similar to that of our cohort. In patients undergoing cardiac surgery, AKI identified by oliguria alone was associated with increased rate of persistent kidney dysfunction at 180 days.

Our results are also comparable with data from the Finnish Acute Kidney Injury (FINNAKI) cohort,^20^ a multicenter prospective study including 2160 patients, although it relied on modified KDIGO criteria to define oliguria. In that study, oliguria (UO <0.5 mL/kg/h) was associated with a significantly higher 90-day mortality only if an episode lasted more than 12 hours. Lower thresholds were explored and more severe episodes (UO <0.1 mL/kg/h) lasting for more than 3 hours were also found to be associated with mortality.

### Implications for Clinicians and Policy Makers

Our data suggest that oliguria-based criteria for AKI diagnosis and classification, as proposed by the current KDIGO consensus, improve the diagnostic performance of sCr-based criteria used alone. Oliguria-based criteria identify AKI in patients without any elevation of sCr levels and reclassify several AKI cases based on sCr criteria to higher AKI stages. Such identifications and reclassifications have significant prognostic implications at least for stages 2 and 3 AKI. Hence, oliguria-based criteria improve patients’ risk estimation and clinicians’ ability to evaluate the burden of AKI.

Oliguria-based parameters should therefore remain included in further iterations of AKI diagnostic criteria. Similarly, future studies should consider oliguria-based criteria to define AKI and not solely rely on sCr changes. Perhaps the implementation of electronic flow measurement devices could facilitate the collection of such data and improve the reliability and accuracy of UO measurement. Finally, clinicians at the bedside should be aware of the long-term implications of oliguria, irrespective of changes in sCr level.

### Strengths and Limitations

Our study has several strengths. First, it included a large number of observations (15 620 patients) corresponding to most patients admitted to our ICU during the study period, with the main exception of those who declined to participate in research. Second, UO values were collected prospectively in the context of routine patient monitoring. The very low rate of missing values (median number of missing values, 5.4% per patient) suggests a high level of reliability. In addition, our sensitivity analyses confirmed that missing UO values did not influence our results. Based on those data, we could compute urinary parameters using unmodified KDIGO criteria, considering its timing component, as thoroughly described in the Methods section. Third, patients’ survival was assessed by cross-referencing our database with the Swiss national death registry, a very reliable source. This method also enabled extended follow-up durations.

Nonetheless, our study has limitations that are worth discussing. First, as a monocentric study, its external validity might be questioned. However, patients’ characteristics, the incidence of AKI, and the UO contribution in our study are largely consistent with data from previous publications in other health care environments.^6,21,27,28,29,30^ Second, as a retrospective study, our conclusions are inherently limited to the description of potential associations between oliguria and adverse outcomes but cannot validate causal relations. However, given the granularity of our database and the number of patients, we were able to include numerous parameters in our multivariate analyses and account for most of the major known cofounders. Third, almost half our patients did not have a true baseline sCr value, a major determinant of KDIGO stage. For these patients, we considered the lowest value obtained during the index hospital admission (outside periods of KRT). This can be challenged because sCr values are known to undergo slow decreases during prolonged ICU stays (due to a loss of lean body mass).^31^ However, attributed values differed marginally from measured values (Table 1). More importantly, sensitivity analyses strongly suggest that such imputations had no impact on overall results (eFigure 5 in the Supplement). Fourth, our decision to choose the lowest sCr measurement in the prior 12 months as the reference baseline sCr level can be challenged because this may lead to bidirectional misclassification of AKI and affect its incidence and mortality.^32^ However, alternative definitions commonly found in the literature^32^ were assessed through sensitivity analyses and did not appear to influence our conclusions (eFigure 4 in the Supplement).

Fifth, UO measurements were manually reported on an hourly basis in our electronic medical records by nurses at the bedside. Undeniably, transcription errors might have occurred^33^; however, the strength and robustness of the observed associations and the high number of observations reduce the burden of such errors. In addition, the high nurse to patient ratio in our ICU (1:1.2) is likely to minimize transcription and aggregation errors. Last, according to the current KDIGO recommendations, UO has been normalized by patients’ body weight. This parameter is known to be highly inaccurate because it is influenced by many external factors such as volume status, vasopressors, or diuretics, which were not accounted for in this study. This potential bias was minimized by the sole consideration of preadmission weight if available. This value was missing in 3206 patients (20.5%). Again, our sensitivity analyses do not suggest that our imputations had a major influence on our results.

## Conclusions

In this cohort study, oliguria lasting more than 12 hours (KDIGO stage 2 or 3) was associated with 90-day mortality irrespective of concomitant changes in sCr levels relative to baseline. Episodes of shorter duration (<12 hours) did not carry similar significance.

## Notes

Supplement. **eTable 1.** Between-Groups Outcomes Comparisons **eTable 2.** Missing Values **eTable 3.** Sensitivity Analyses: Multivariate Logistic Regression Model Without SAPS II Score **eFigure 1.** Overall Survival Probability in No AKI, UO-Only, sCr-Only, and sCr Plus UO Groups **eFigure 2.** Predictive Margins of 90-Day Mortality According to sCr and UO KDIGO Stages **eFigure 3.** Sensitivity Analyses: Missing Values **eFigure 4.** Sensitivity Analyses: Alternative Baseline sCr Definitions **eFigure 5.** Sensitivity Analyses: Alternative Baseline sCr Imputation Method
