# Supplementary material for: Association of Oliguria With Acute Kidney Injury Diagnosis, Severity Assessment, and Mortality Among Patients With Critical Illness
Source: JAMA Netw Open. 2021 Nov 4;4(11):e2133094. doi: 10.1001/jamanetworkopen.2021.33094 (PMC8569487; doi:10.1001/jamanetworkopen.2021.33094)
Supplement: Supplement. — eTable 1. Between-Groups Outcomes Comparisons eTable 2. Missing Values eTable 3. Sensitivity Analyses: Multivariate Logistic Regression Model Without SAPS II Score eFigure 1. Overall Survival Probability in No AKI, UO-Only, sCr-Only, and sCr Plus UO Groups eFigure 2. Predictive Margins of 90-Day Mortality According to sCr and UO KDIGO Stages eFigure 3. Sensitivity Analyses: Missing Values eFigure 4. Sensitivity Analyses: Alternative Baseline sCr Definitions eFigure 5. Sensitivity Analyses: Alternative Baseline sCr Imputation Method [file jamanetwopen-e2133094-s001.pdf]

Supplementary Online Content

Bianchi NA, Stavart LL, Altarelli M, Kelevina T, Faouzi M, Schneider AG. Association of oliguria with acute kidney injury diagnosis, severity assessment, and mortality among patients with critical illness. *JAMA Netw Open*. 2021;4(11):e2133094. doi:10.1001/jamanetworkopen.2021.33094

eTable 1. Between-Groups Outcomes Comparisons
eTable 2. Missing Values
eTable 3. Sensitivity Analyses: Multivariate Logistic Regression Model Without SAPS II Score
eFigure 1. Overall Survival Probability in No AKI, UO-Only, sCr-Only, and sCr Plus UO Groups
eFigure 2. Predictive Margins of 90-Day Mortality According to sCr and UO KDIGO Stages
eFigure 3. Sensitivity Analyses: Missing Values
eFigure 4. Sensitivity Analyses: Alternative Baseline sCr Definitions
eFigure 5. Sensitivity Analyses: Alternative Baseline sCr Imputation Method

This supplementary material has been provided by the authors to give readers additional information about their work.

© 2021 Bianchi NA et al. *JAMA Network Open*.

eTable 1. Between-Groups Outcomes Comparisons

Outcome	"No AKI" vs "UO only"	"No AKI" vs "sCr only"	"No AKI" vs "sCr plus UO"	"UO only" vs "sCr only"	"UO only" vs "sCr plus UO"	"sCr only" vs "sCr plus UO"
ICU LOS, days, median (IQR)	< 0.001	< 0.001	< 0.001	< 0.001	< 0.001	< 0.001
Hospital LOS, days, median (IQR)	< 0.001	< 0.001	< 0.001	< 0.001	< 0.001	< 0.001
RRT during ICU stay, n (%)	-[a]	< 0.001	< 0.001	< 0.001	< 0.001	< 0.001
Mechanical ventilation (MV), n (%)	< 0.001	0.005	< 0.001	0.011	< 0.001	< 0.001
MV total duration, hours, median (IQR)	< 0.001	< 0.001	< 0.001	< 0.001	< 0.001	< 0.001
ICU mortality, n (%)	< 0.001	0.001	< 0.001	0.922	< 0.001	< 0.001
Hospital mortality, n (%)	< 0.001	< 0.001	< 0.001	0.020	< 0.001	< 0.001
90-Day mortality, n (%)	< 0.001	< 0.001	< 0.001	< 0.001	< 0.001	< 0.001
1-Year mortality, n (%)	< 0.001	< 0.001	< 0.001	< 0.001	< 0.001	< 0.001
3-Year mortality, n (%)	< 0.001	< 0.001	< 0.001	< 0.001	< 0.001	< 0.001
5-Year mortality, n (%)	< 0.001	< 0.001	< 0.001	< 0.001	< 0.001	0.026

Reported p-values refer to one-to-one comparisons between respective groups using Chi-square and Wilcoxon Rank-sum tests for categorical and continuous variables respectively. P-values <0.001 were considered statistically significant.

[a] For “no AKI” and “UO only” groups, none of the patients received RRT, therefore comparison could not be made.

© 2021 Bianchi NA et al. *JAMA Network Open*.

eTable 2. Missing Values

Parameter	Missing values, n (%)
Age at ICU admission	0 (0)
Gender	0 (0)
Actual body weight[a]	3'206 (20.5)
Actual baseline creatinine[a]	7'547 (48.3)
Creatinine at ICU admission	0 (0)
Daily serum creatinine[b]	9’641 (9.8)
Hourly urinary output[c]	280’990 (14.4)
SAPS II score	0 (0)
Co-existing conditions & Charlson score	49 (0.3)
Type of admission *(Surgical vs. Medical)*	360 (2.3)
Elective admission	360 (2.3)
Mechanical ventilation within first 24 hours	0 (0)
Noradrenaline within first 24 hours	0 (0)
Main ICU diagnosis	415 (2.7)

ICU, intensive care unit; SAPS, simplified acute physiology score; MDRD, modification of diet in renal disease; eGFR, estimated glomerular filtration rate.

[a] Imputations were performed as described in the methods section.
[b] Median percentage (interquartile range) of missing values per patient: 0.0 (0.0, 25.0) days,
[c] Median percentage (interquartile range) of missing values per patient 5.4 (2.4, 16.7) hours.

© 2021 Bianchi NA et al. *JAMA Network Open*.

eTable 3. Sensitivity Analyses: Multivariate Logistic Regression Model Without SAPS II Score

	Multivariate analysis		
**Model including SAPS II score (main model)**			
**Max AKI Stage - UO criteria**	**OR**	**95% CI**	**p-value**
No AKI	Reference	-	-
Stage 1	1.32	(0.76 – 2.26)	0.322
Stage 2	2.43	(1.57 – 3.77)	<0.001
Stage 3	6.24	(3.70 – 10.52)	<0.001
**Max AKI Stage - sCr criteria**			
No AKI	Reference	-	-
Stage 1	1.15	(1.02 – 1.31)	0.029
Stage 2	1.32	(1.11 – 1.57)	0.002
Stage 3	1.73	(1.45 – 2.07)	<0.001
**Model without SAPS II score**			
**Max AKI Stage - UO criteria**	**OR**	**95% CI**	**p-value**
No AKI	Reference	-	-
Stage 1	1.36	(1.16 – 1.60)	<0.001
Stage 2	1.38	(1.21 – 1.57)	<0.001
Stage 3	2.77	(2.33 – 3.29)	<0.001
**Max AKI Stage - sCr criteria**			
No AKI	Reference	-	-
Stage 1	1.45.	(1.29 – 1.64)	<0.001
Stage 2	1.94	(1.65 – 2.29)	<0.001
Stage 3	2.93	(2.48 – 3.46)	<0.001

Odds ratios and 95% confidence interval for 90-day mortality are represented for each KDIGO stage.
Model with SAPS includes: modified SAPS II score (renal parameters excluded), age, baseline sCr, Charlson score, main ICU diagnosis, and both sCr and UO-based AKI stages according to KDIGO classification.
Model without SAPS includes: age, baseline sCr, Charlson score, main ICU diagnosis, and both sCr and UO-based AKI stages according to KDIGO classification.
OR, Odd Ratio; CI, Confidence Interval; UO, Urinary Output; sCr, Serum Creatinine.

© 2021 Bianchi NA et al. *JAMA Network Open*.

eFigure 1. Overall Survival Probability in No AKI, UO Only, sCr Only, and sCr Plus UO Groups

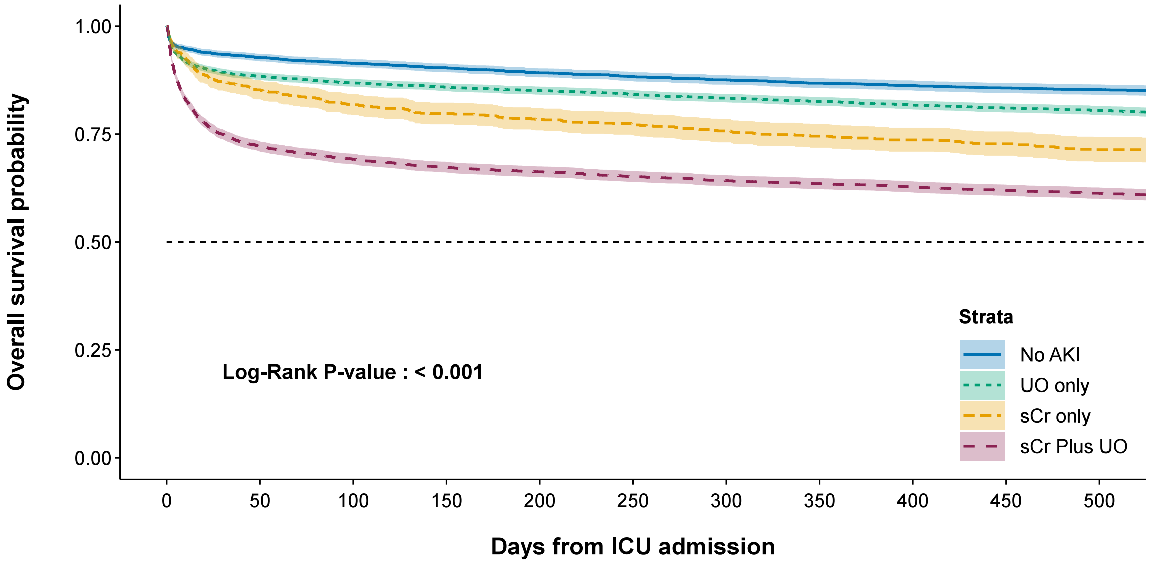

Number at risk (%)

No AKI	3477 (100)	3224 (93)	3155 (91)	3094 (89)	3019 (87)	2955 (85)	2892 (83)	2830 (81)	2774 (80)	2717 (78)	2660 (77)
UO only	5630 (100)	4978 (88)	4858 (86)	4743 (84)	4628 (82)	4534 (81)	4423 (79)	4324 (77)	4235 (75)	4150 (74)	4067 (72)
sCr only	989 (100)	843 (85)	800 (81)	776 (78)	749 (76)	728 (74)	705 (71)	677 (68)	660 (67)	640 (65)	621 (63)
sCr Plus UO	5524 (100)	3984 (72)	3798 (69)	3630 (66)	3529 (64)	3425 (62)	3325 (60)	3230 (58)	3146 (57)	3062 (55)	2992 (54)

Kaplan-Meier curves with 95% CI representing the estimated survival probability over 500 days after ICU admission for the “No AKI”, “UO only”, “sCr only” and “sCr Plus UO” groups. Overall as well as one-to-one comparisons between survival curves were performed using log-rank tests, all p-values <0.001.
CI: Confidence interval, sCr: Serum creatinine, UO: Urinary output.

© 2021 Bianchi NA et al. *JAMA Network Open*.

eFigure 2. Predictive Margins of 90-Day Mortality According to sCr and UO KDIGO Stages

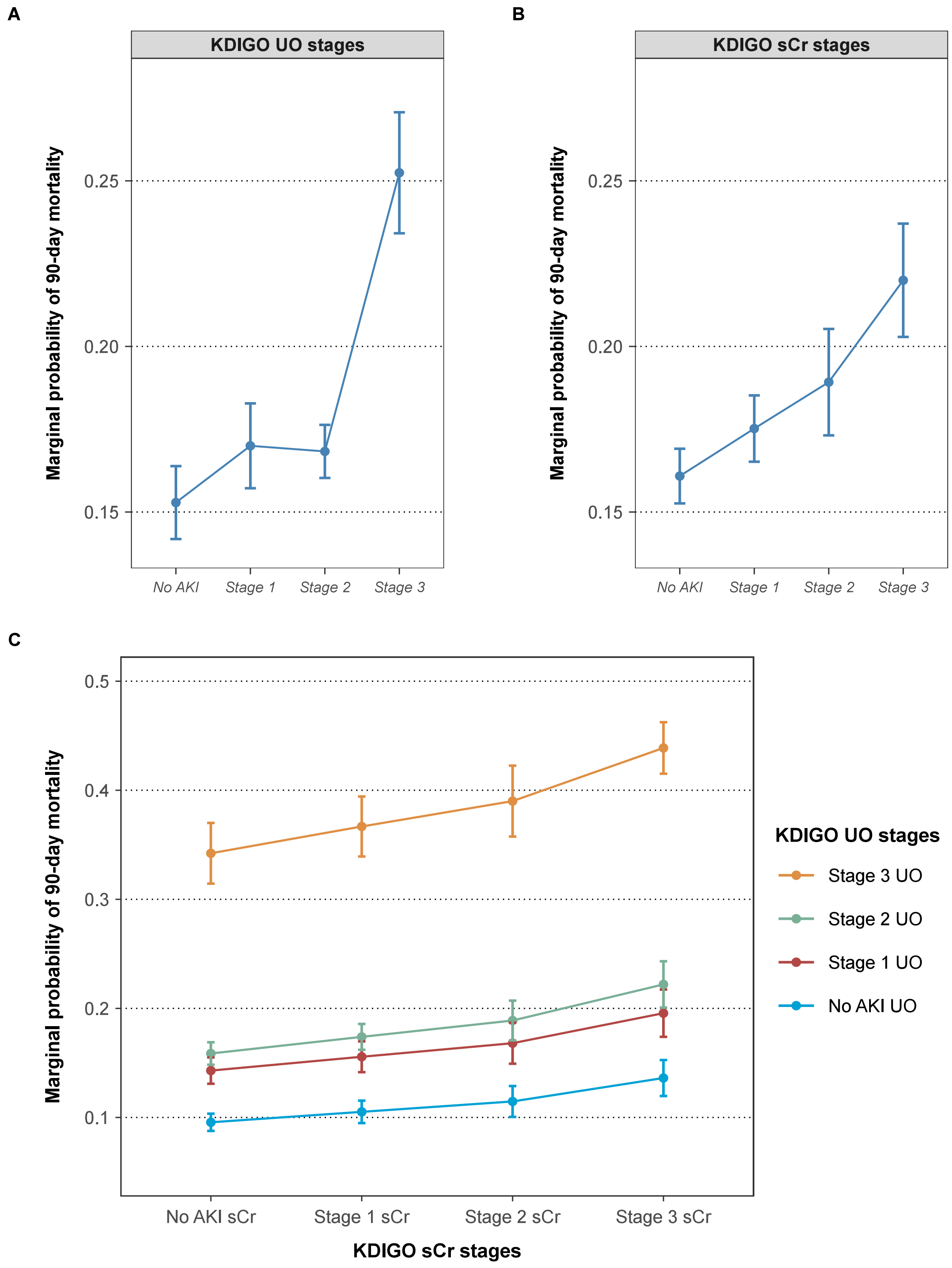

A. Marginal probability (with 95% confidence intervals) of 90-day mortality according to KDIGO sCr stage, adjusted for KDIGO UO stage, age, baseline sCr, main ICU diagnoses, Charlson score, SAPS II score.
B. Marginal probability (with 95% confidence intervals) of 90-day mortality according to KDIGO UO stage, adjusted for KDIGO sCr stage, age, baseline sCr, main ICU diagnoses, Charlson score, SAPS II score.
C. Marginal probability (with 95% confidence intervals) of 90-day mortality represented for each combination of AKI KDIGO sCr and UO stages criteria, adjusted for age, baseline sCr, main ICU diagnoses, Charlson score, SAPS II score. No interactions were found between sCr and UO criteria.

© 2021 Bianchi NA et al. *JAMA Network Open*.

eFigure 3. Sensitivity Analyses: Missing Values

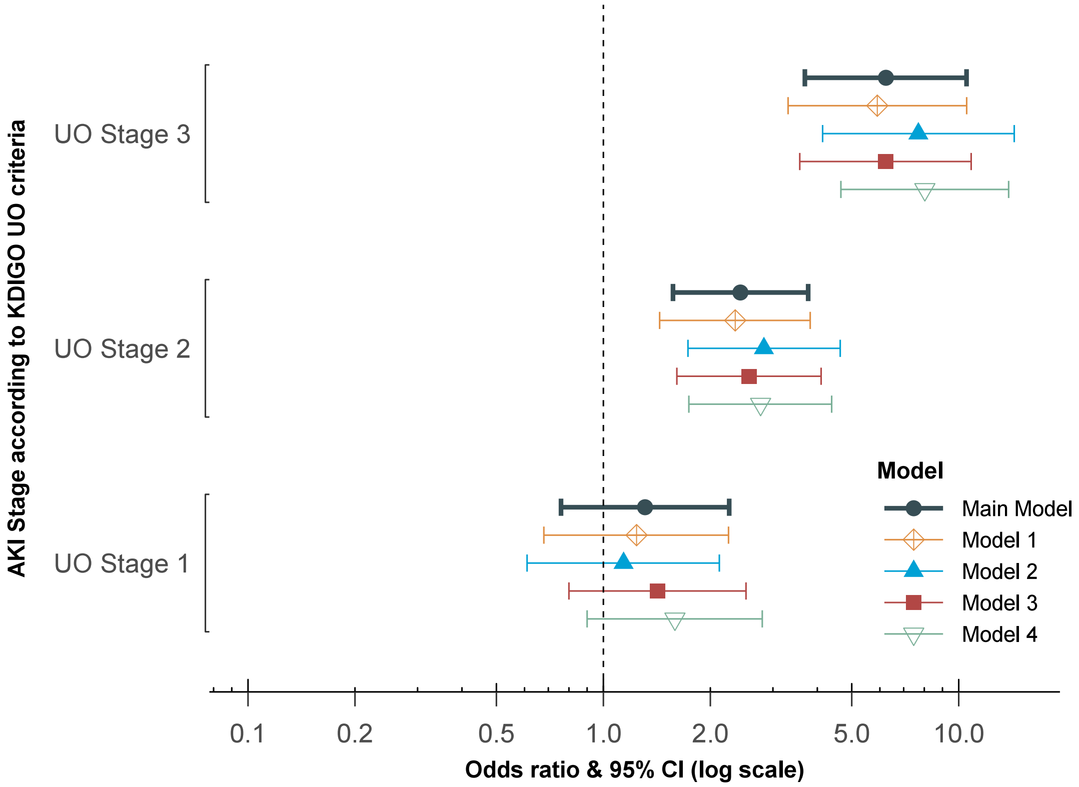

Odds ratios and 95% confidence interval for 90-day mortality are represented for each KDIGO UO stage, according to the different models. All models include age, baseline sCr, modified SAPS II score, Charlson score, main ICU diagnosis, and sCr-based AKI stage according to KDIGO. In addition, model 1-4 include interactions between UO stages and (1) baseline sCr (known vs. inferred) (**Model 1**), (2) body weight (known vs. inferred) (**Model 2**), (3) absence of imputed daily sCr values (yes vs. no) (**Model 3**), and (4) < median percentage (5.4%) of imputed hourly values (yes vs. no) (**Model 4**). For all analyses, a two-tailed p-value < 0.05 was considered statistically significant. For detailed description of the sensitivity analysis, please refer to the methods section.
UO: Urinary Ouput, AKI: Acute Kidney Injury, CI: Confidence Interval

© 2021 Bianchi NA et al. *JAMA Network Open*.

eFigure 4. Sensitivity Analyses: Alternative Baseline sCr Definitions

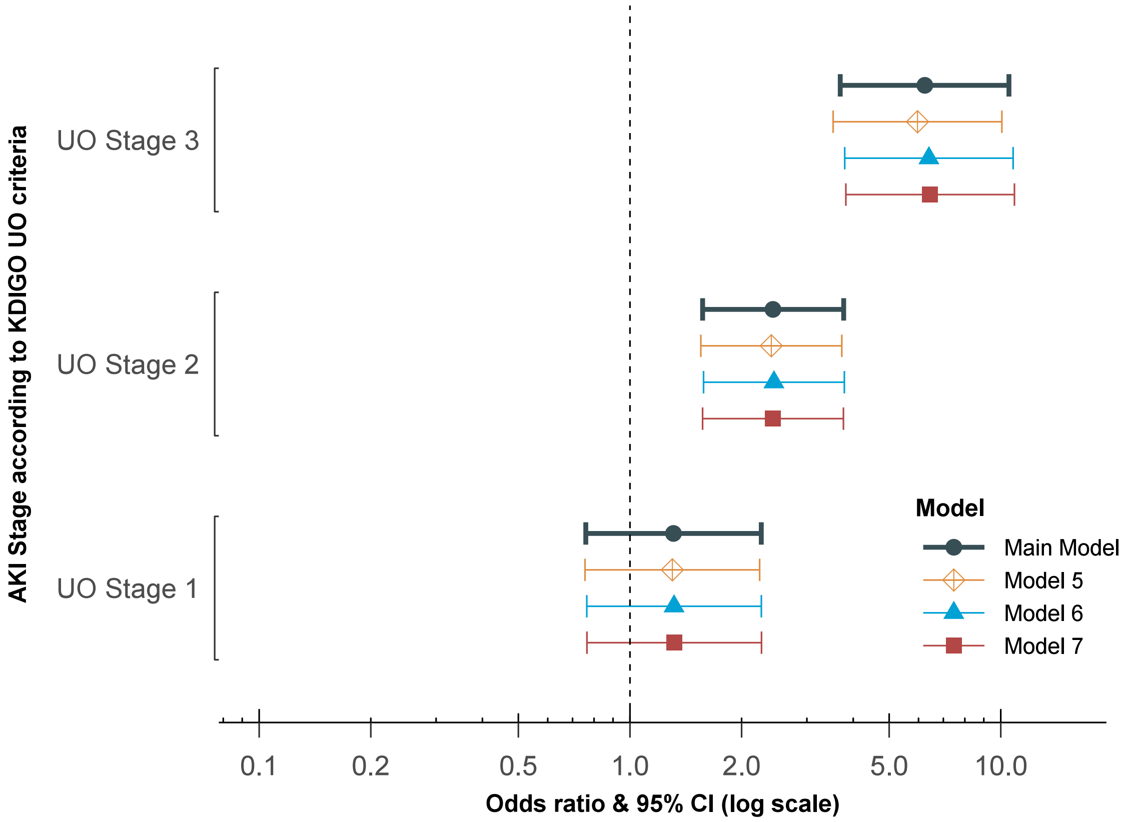

Odds ratios and 95% confidence interval for 90-day mortality are represented for each KDIGO UO stage, according to the different models. All models include age, baseline sCr, modified SAPS II score, Charlson score, main ICU diagnosis, and sCr-based AKI stage according to KDIGO. In addition to the main sCr baseline definition (please refer to the methods section of the manuscript), three alternative definitions were used to diagnose and stage AKI : (1) mean sCr over the 365 days prior to ICU admission (**Model 5**), (2) mean sCr over the 1 to 365 days prior to ICU admission (values measured within 24 hours prior to ICU admission excluded) (**Model 6**) and (3) mean sCr over the 7 to 365 days preceding ICU admission (values measured within 7 days prior to ICU admission excluded) (**Model 7**). For all analyses, a two-tailed p-value < 0.05 was considered statistically significant. For detailed description of the sensitivity analysis, please refer to the methods section.
UO: Urinary Ouput, AKI: Acute Kidney Injury, CI: Confidence Interval

© 2021 Bianchi NA et al. *JAMA Network Open*.

eFigure 5. Sensitivity Analyses: Alternative Baseline sCr Imputation Method

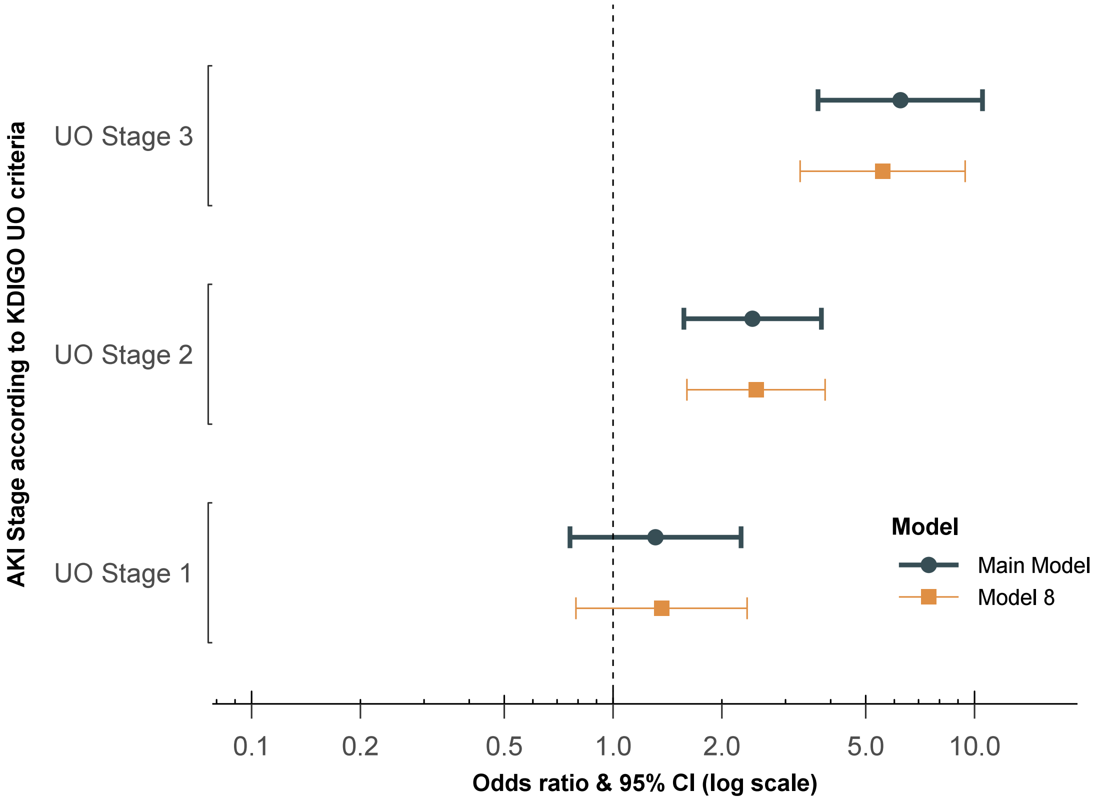

Odds ratios and 95% confidence interval for 90-day mortality are represented for each KDIGO UO stage, according to the different models. All models include age, baseline sCr,modified SAPS II score, Charlson score, main ICU diagnosis, and sCr-based AKI stage according to KDIGO. In case of missing sCr baseline, two sCr baseline imputation methods to diagnose and stage AKI were used : (1) lowest sCr value within the ICU stay (values measured during RRT excluded) (**Main model**), and (2) sCr baseline estimation using MDRD formula based on patient's age and sex, assuming a normal gfr of 75 mL/min/1.73m^2 (**Model 8**).For all analyses, a two-tailed p-value < 0.05 was considered statistically significant. For detailed description of the sensitivity analysis, please refer to the methods section.
UO: Urinary Ouput, AKI: Acute Kidney Injury, CI: Confidence Interval

© 2021 Bianchi NA et al. *JAMA Network Open*.
